# Chondroitin sulfates in the developing rat hindbrain confine commissural projections of vestibular nuclear neurons

**DOI:** 10.1186/1749-8104-7-6

**Published:** 2012-02-03

**Authors:** Jessica CF Kwok, Ying-Lai Yuen, Wai-Kit Lau, Fu-Xing Zhang, James W Fawcett, Ying-Shing Chan, Daisy KY Shum

**Affiliations:** 1 Department of Biochemistry, LKS Faculty of Medicine, The University of Hong Kong, Sassoon Road, Hong Kong, China; 2 Cambridge Centre for Brain Repair, Cambridge University, Forvie Site, Robinson Way, Cambridge CB2 0PY, UK; 3 Department of Physiology, LKS Faculty of Medicine, The University of Hong Kong, Sassoon Road, Hong Kong, China; 4 Department of Anatomy, Histology and Embryology, The Fourth Military Medical University, Xi'an, China; 5 Research Centre of Heart, Brain, Hormone and Healthy Aging, LKS Faculty of Medicine, The University of Hong Kong, Sassoon Road, Hong Kong, China

## Abstract

**Background:**

Establishing correct neuronal circuitry is crucial to proper function of the vertebrate nervous system. The abundance of chondroitin sulfate (CS) proteoglycans in embryonic neural environments suggests that matrix proteoglycans regulate axonal projections when fiber tracts have not yet formed. Among the early-born neurons, the vestibular nucleus (VN) neurons initiate commissural projections soon after generation at E12.5 and reach the contralateral target by E15.5 in the rat hindbrain. We therefore exploited 24-hour cultures (1 day *in vitro *(DIV)) of the rat embryos and chondroitinase ABC treatment of the hindbrain matrix to reveal the role of CS moieties in axonal initiation and projection in the early hindbrain.

**Results:**

DiI tracing from the VN at E12.5_(+1 DIV) _showed contralaterally projecting fibers assuming fascicles that hardly reached the midline in the controls. In the enzyme-treated embryos, the majority of fibers were unfasciculated as they crossed the midline at 90°. At E13.5_(+1 DIV)_, the commissural projections formed fascicles and crossed the midline in the controls. Enzyme treatment apparently did not affect the pioneer axons that had advanced as thick fascicles normal to the midline and beyond, towards the contralateral VN. Later projections, however, traversed the enzyme-treated matrix as unfasciculated fibers, deviated from the normal course crossing the midline at various angles and extending beyond the contralateral VN. This suggests that CSs also limit the course of the later projections, which otherwise would be attracted to alternative targets.

**Conclusions:**

CS moieties in the early hindbrain therefore control the course and fasciculation of axonal projections and the timing of axonal arrival at the target.

## Background

The establishment of correct neuronal circuitry is crucial for proper function of the vertebrate nervous system. During development of the vertebrate hindbrain, the early but transient subdivision of the neuroepithelium into rhombomeres [1,2] lays the scaffold for the development of the complex nervous system. Studies have focused on identifying the intercellular signals and transcription factors involved in defining and maintaining the cell identity of individual rhombomeres [3-6]. Less is known about the mechanisms that regulate cell movements important for the patterning and morphogenesis of the hindbrain.

The vestibular nucleus (VN) is generated as four main neuronal clusters, each spanning several rhombomeres but differing in the rostrocaudal span along the hindbrain [7-10]. Radiographic birth-dating indicated an orderly progression led by neurons of the lateral VN, then the superior VN and inferior VN, and finally the medial VN, with peaks respectively on embryonic day (E)12.5 to E14.5 in the rat [7]. Afferent projections from these nuclei to major vestibulo-motor-related targets were observable as early as E11.5 (mouse) and E12.5 (rat) [7-10]. Once the pioneer afferents have been initiated, the advancing growth cones navigate the 'local' environment for positional and directional cues in the determination of a path to their target. Little is known about molecules involved in the pathway navigation. Studies on other systems indicated involvement of the Eph receptor tyrosine kinases and their membrane-bound ligand, ephrins, in growth cone negotiation with rhombomere boundaries [11-13]. The chondroitin sulfate (CS) proteoglycans (CSPGs) are strategically located in the extracellular matrix of the developing brain to influence cell-matrix adhesion and neurite outgrowth. In the chick brain, expression of CS was found to peak at the rhombomeric stage of hindbrain development [14]. Despite reports of CSPGs in the developing brain, their role in fiber projections in early embryos when pioneer axons are negotiating the path to reach their targets remains largely unexplored.

In this study, we are the first to report live treatment of the hindbrain matrix of rat embryos (E12.5 to E14.5) with chondroitinase ABC (Ch^ABC^) for 1 day in culture during which the course of commissural projections is perturbed by enzymatic depletion of CS moieties. Following the treatment, we employed the lipophilic dye DiI to track contralateral projections from the VN in fixed hindbrains. The consequent increases in projection distance and number of unfasciculated fibers as well as the deviated courses of unfasciculated fibers revealed that axon-restrictiveness of CS moieties in the early hindbrain matrix controls the timing and abundance of axons that end at the contralateral VN target.

## Materials and methods

### Animal sources

Pregnant Sprague Dawley rats (gestation day 12.5 to 14.5; n ≥ 5 for each stage) were provided by the Laboratory Animal Unit at The University of Hong Kong. All procedures were in strict accordance with the NIH Guide for the Care and Use of Laboratory Animals and approved by the Committee on the Use of Live Animals for Teaching and Research (The University of Hong Kong).

Pregnant rats were anaesthetized with sodium pentobarbital (30 mg/kg) and the abdomen dissected for embryo collection. After transfer of embryos to DMEM (Sigma, St. Louis, MO, USA), the pregnant rats were sacrificed with an overdose of sodium pentobarbital (60 mg/kg).

### Embryo culture and chondroitinase ABC injection

**Figure 1:**
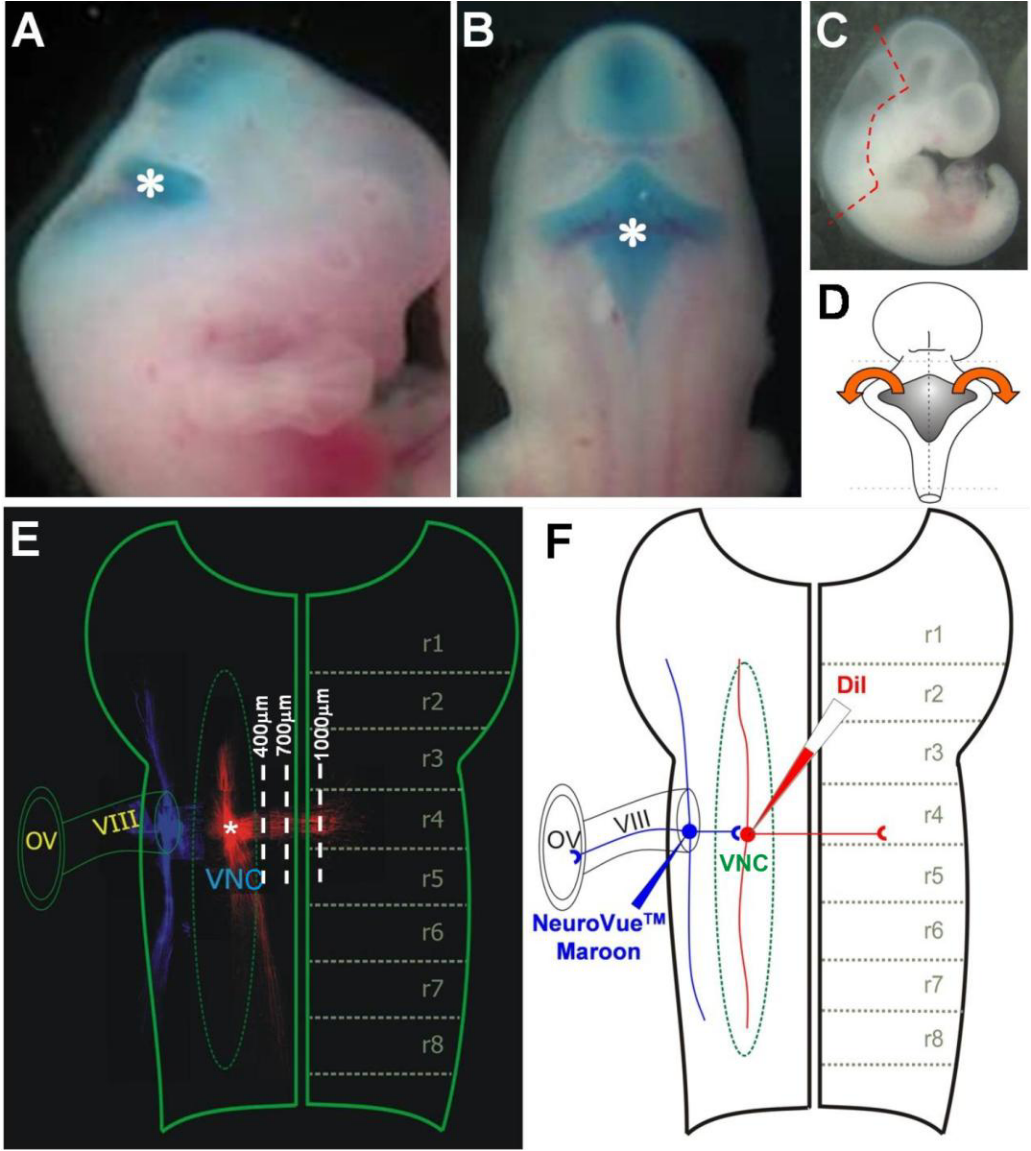
**Preparation of the embryo**. **(A,B) **Injection of Ch^ABC ^into the fourth ventricle in the embryos was visualized by fast green included in the enzyme solution: (A) side view and (B) dorsal view of the embryos. The asterisk marks the position of the fourth ventricle. **(C) **The dashed red line outlines the hindbrain region to be dissected out from the embryo. **(D) **Schematic diagram showing cuts made in the dorsal neural tube to expose the ventricular surface of the hindbrain. **(E) **Flat-mounted hindbrain showing the DiI (red) and NeuroVue Maroon (blue) tracked fibers. Overlay of longitudinal gridlines (white dashed lines) assist fiber counting. The center of the dye injection (indicated by the asterisk) was designated as 0 μm. Gridlines of 100 μm were drawn parallel to the floor plate. **(F) **Schematic diagram showing a flat-mounted hindbrain with the ventricular surface facing up. The dashed outline in green indicates the extent of the VN complex (VNC) in the hindbrain. The dotted lines in black indicate the rhombomere boundaries. Targeting of DiI (red) to the VN neurons was confirmed by afferents that were labeled by NeuroVue Maroon (blue) introduced into the VIIIth cranial nerve root and their termination on the DiI-labeled VN neurons. Abbreviations: OV, otic vesicle; r, rhombomere; VIII, VIIIth cranial nerve.

Whole embryo culture was performed as described by New [15]. The decidual tissue and Reichert's membrane were excised from the collected embryos, leaving the intact yolk sac behind. The yolk sac was opened with caution not to damage the yolk-sac vasculature. Ch^ABC ^(1 μl of 10 U/ml; *Proteus vulgaris*, Seikagaku, Tokyo, Japan) was then injected into the fourth ventricle of the embryo. Fast Green (1:10,000 w/v; Sigma) was included in the Ch^ABC ^solution to aid visualization (Figure 1A,B). PBS and heat-inactivated Ch^ABC ^were both used as controls. As the results for both controls were similar, only the results from PBS-treated embryos are shown. The embryos were maintained in culture in DMEM/F12 with 10% fetal calf serum (Gibco, Invitrogen {Grand Island, NY, USA), 100 U/ml of penicillin (Sigma) and 0.1 mg/ml of streptomycin (Sigma) for 24 hours at 37°C with shaking. At the end of the culture period, the embryos were examined for heartbeat and expected increase in size. Only viable embryos were further processed for DiI labeling (Molecular Probes, Invitrogen, Eugene, OR, USA).

### DiI labeling

Hindbrains were dissected out from viable embryos recovered from the culture. Cutting along the dorsal midline allowed flat-mounting of the hindbrain with the ventricular side facing up (Figure 1C,D). The hindbrains were kept thinly overlaid with 4% paraformaldehyde for 24 hours. The lipophilic indocarbocyanine dye DiI (1 mg/ml in DMSO; Molecular Probes, Invitrogen) was applied into the floor of the fourth ventricle to target VN neurons at the rhombomere 4/5 border. To confirm the location of VN neurons, another lipophilic dye, NeuroVue Maroon (Polysciences, Warrington, PA, USA) [16] was applied to the VIIIth cranial nerve root such that the labeled afferent to the VN could be visualized (Figure 1F). After labeling, the hindbrain flat-mounts were kept in 4% paraformaldehyde (Sigma) at 37°C in the dark for 3 days to allow for the diffusion of the dye along fiber tracts. The hindbrains were mounted on glass slides with 1X PBS for observation. Images of labeled cells and fiber outgrowths were captured under a fluorescence microscope Axiophot 2 (Carl Zeiss, Thornwood, NY, USA) or MRC-1024 Laser Scanning Confocal Imaging System (Bio-Rad, Hercules, CA, USA). Montages of the images were constructed.

### Immunohistochemistry

Hindbrains were fixed with 4% paraformaldehyde in 1× PBS at 4°C overnight followed by immersion in 30% sucrose solution for at least 24 hours at 4°C for cryo-protection. Transverse sections (14 μm) were prepared with the cryostat and then incubated (4°C for 16 hours) with the monoclonal antibody CS-56 (1:250; IgM, Sigma) or one of the stub-sensitive antibodies 1B5, 2B6 (1:250; IgG, Seikagaku) or 3B3 (1:250; IgM, Seikagaku) in 1× PBS with 0.3% Triton-X and 3% normal goat serum. CS-56-stained sections were incubated with biotinylated goat anti-mouse IgM secondary antibody (1:500; Molecular Probes, Invitrogen) and then with Streptavidin-Alexa 488 (1:500; Molecular Probes, Invitrogen) for 1 hour at 24°C. Those stained for the stub epitope were incubated with Alexa fluor 488-conjugated donkey anti-mouse IgG (1:500; Molecular Probes, Invitrogen). The sections were coverslipped and images observed under a Leica Leitz DMRD microscope were captured with a Nikon Digital Camera DMX1200.

### Quantification of DiI-labeled fibers

Montage images from both PBS- (n > 5) and Ch^ABC^-treated hindbrains (n > 5) were assessed for number of contralaterally projecting fibers and their state of fasciculation. Fibers were considered unfasciculated when diameters were ≤ 5 μm and fasciculated when diameters were > 5 μm. For fiber counting, a grid of longitudinal lines spaced at 100 μm intervals was placed against the montage and the DiI-injection site was set as zero. Fasciculated and unfasciculated fibers that crossed each gridline were counted.

### Statistical analysis

All data are expressed as means ± standard deviation. Data resulting from Ch^ABC ^treatment versus PBS controls were analyzed by the Mann-Whitney's U test with use of the computer software PRISM (GraphPad Software, Inc La Jolla, CA, USA **{**). Statistical significance was accepted at *P *< 0.05.

## Results

### Early embryos survive Ch^ABC ^treatment of the hindbrain matrix

**Figure 2:**
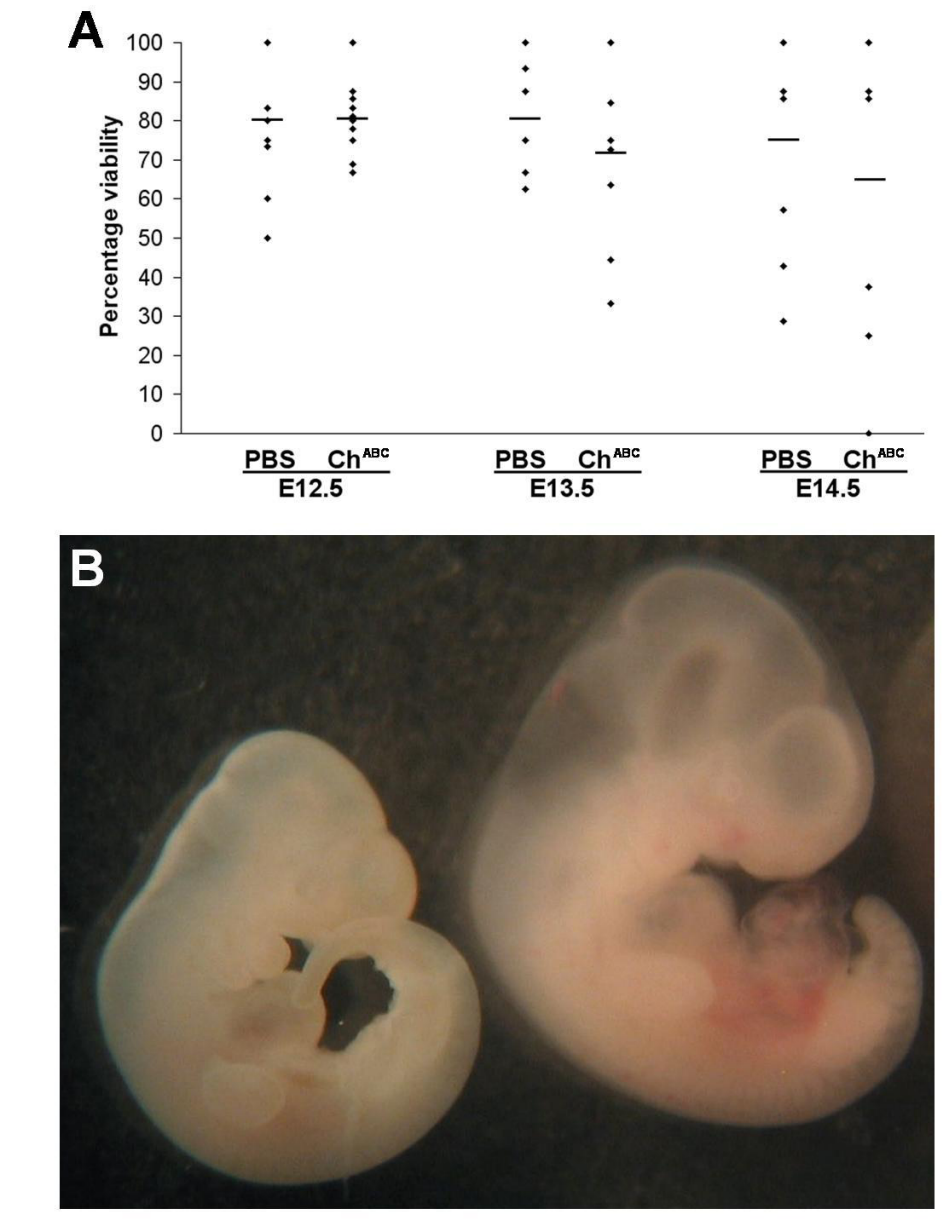
**Viability of embryos in 24-hour culture with PBS/Ch^ABC ^delivered to the fourth ventricle**. **(A) **Plot of percentage viability against the indicated stage and treatment. Each dot represents the percentage viability of the litter tested. The data were analyzed using the two-tailed Student's *t*-test. **(B) **E12.5 embryo (left) compared to an E12.5_(+1 DIV) _(right) litter mate, showing significant growth in size after 24 hours in culture.

To determine if Ch^ABC ^treatment via the fourth ventricle affected viability of the embryos in *ex vivo *culture, the embryos were rated as viable if heartbeat and growth in body size at the end of the 24-hour culture were comparable to those of similar stage embryos freshly harvested from the mother. Embryos that did not measure up to these criteria were excluded from the study. As shown for the viable embryo at E12.5 versus E12.5_(+ 1 DIV) _(Figure 2B), growth in size was obvious. In all stages studied, the number of somites in viable embryos that survived Ch^ABC ^treatment was the same as that in the PBS controls and that expected at the embryonic stage. Treatment with Ch^ABC ^did not differ significantly from the PBS control in viability among embryos at E12.5_(+ 1 DIV)_, averaging 80 ± 10% (Figure 2A). At E13.5_(+ 1 DIV)_, the viability dropped to 71 ± 23% with Ch^ABC ^treatment, significantly lower (*P *< 0.05) than the viability of 82 ± 15% maintained by the PBS controls. At E14.5_(+1 DIV)_, viability dropped further to 65 ± 33% with Ch^ABC ^treatment, not significantly different from the 75 ± 29% of the PBS controls. Taken together, embryo culture at E14.5_(+1 DIV) _is limiting to life, whereas at E13.5_(+1 DIV)_, CS-dependent life processes are compromised by depletion of CS in the hindbrain matrix, and at E12.5_(+1 DIV)_, these CS-dependent life processes have possibly not been fully differentiated, if at all.

### Ch^ABC ^diffuses into the hindbrain matrix and acts to cleave the matrix CS

**Figure 3:**
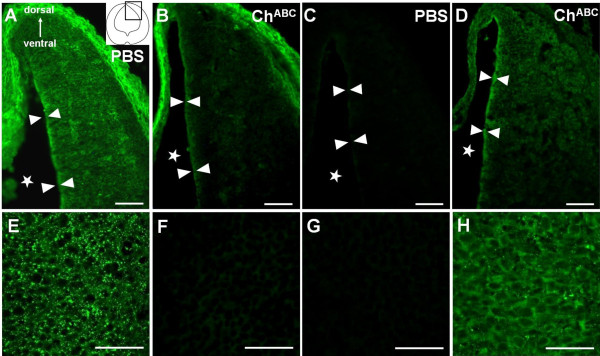
**Immunoreactivity for CS epitopes in hindbrain sections of E13.5_(+1 DIV) _hindbrain sections**. **(A-H) **Staining for the CS-56 epitope following control treatment with PBS (A,B,E,F) and the 2B6-stub epitope following Ch^ABC ^treatment (C,D,G,H). Images correspond to the boxed region shown in the schematic diagram of the hindbrain section in (A). Zoomed-in views of the hindbrain matrix are shown below the corresponding images of the hindbrain sections. Asterisks indicate the region of the fourth ventricle; arrowheads indicate the outline of the ventricular surfaces of the hindbrain. Sections are 14 μm. Scale bars: 100 μm (upper panels); 50 μm (lower panels).

If Ch^ABC ^in the injection mix could digest the hindbrain matrix CS, unsulfated (ΔDi-0S), 4-sulfated (ΔDi-4S) or 6-sulfated (ΔDi-6S) stubs that remain with the matrix proteoglycans would be recognized by the monoclonal antibodies 1B5, 2B6 and 3B3, respectively. Otherwise, the intact CS would stain positive with the monoclonal antibody CS56. As expected, the hindbrain matrix of PBS controls stained positive with CS56 (Figure 3A,E) but showed no immunoreactivity with 2B6 (Figure 3C,G) and likewise with 1B5 and 3B3 (results not shown). With Ch^ABC ^treatment, the hindbrain matrix then lost immunoreactivity with CS56 (Figure 3B,F) and developed immunoreactivity with 2B6 (Figure 3D,H); signals for 1B5 and 3B3 were barely detectable (results not shown). The results therefore indicate that CS moieties of the hindbrain matrix were accessible to digestion by Ch^ABC ^delivered into the fourth ventricle and that CS stubs exposed as a result of Ch^ABC ^activity on the matrix CSPGs were essentially 4-sulfated.

In contrast, strong CS56-positive signal in the mesenchymal tissue bordering the neuroepithelial tissue of the hindbrain (Figure 3A,B) remained strongly CS56-positive after Ch^ABC ^treatment, apparently unperturbed or inaccessible by Ch^ABC ^delivered into the fourth ventricle. Strong CS56-positive signals were also found along the ventricular surface of the hindbrain (PBS-treated; Figure 3A). Following Ch^ABC ^treatment, weakening of these signals (Figure 3B) was accompanied by emergence of strong 2B6-positive signals (Figure 3D). These results indicate that despite proximity of Ch^ABC ^to the ventricular zone, the enrichment of CSPGs and their involvement in cell/matrix interactions render the CS moieties incompletely accessible to Ch^ABC ^activity. Taken together, Ch^ABC ^delivered into the fourth ventricle adequately depleted CS moieties of the hindbrain matrix.

### Changes in commissural projections with depletion of matrix CS moieties

**Figure 4:**
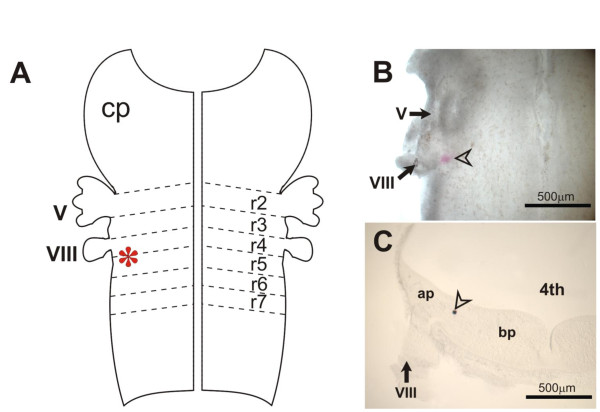
**Preliminary experiments to decide landmarks for DiI application to the VN**. **(A) **Schematic diagram showing a flat-mounted hindbrain with the ventricular surface facing up. DiI application was at a site close to the VIIIth cranial nerve root and along the rhombomere 4/5 border (indicated by the asterisk). **(B) **Brightfield image of a flat-mounted hindbrain (E13.5) after DiI/DMSO was injected at a site (arrowhead) close to the VIIIth cranial nerve root (VIII). **(C) **Transverse section of the hindbrain at the level of the VIIIth cranial nerve root (VIII) after DiI crystal was applied at a site (arrowhead) approximately 75 μm from the floor of the fourth ventricle and in the alar plate. Abbreviations: 4th, fourth ventricle; ap, alar plate; bp, basal plate; cp, cerebellar primordium; V, Vth cranial nerve root; VIII, VIIIth cranial nerve root; r2 to r7, rhombomeres 2 to 7.

**Figure 5:**
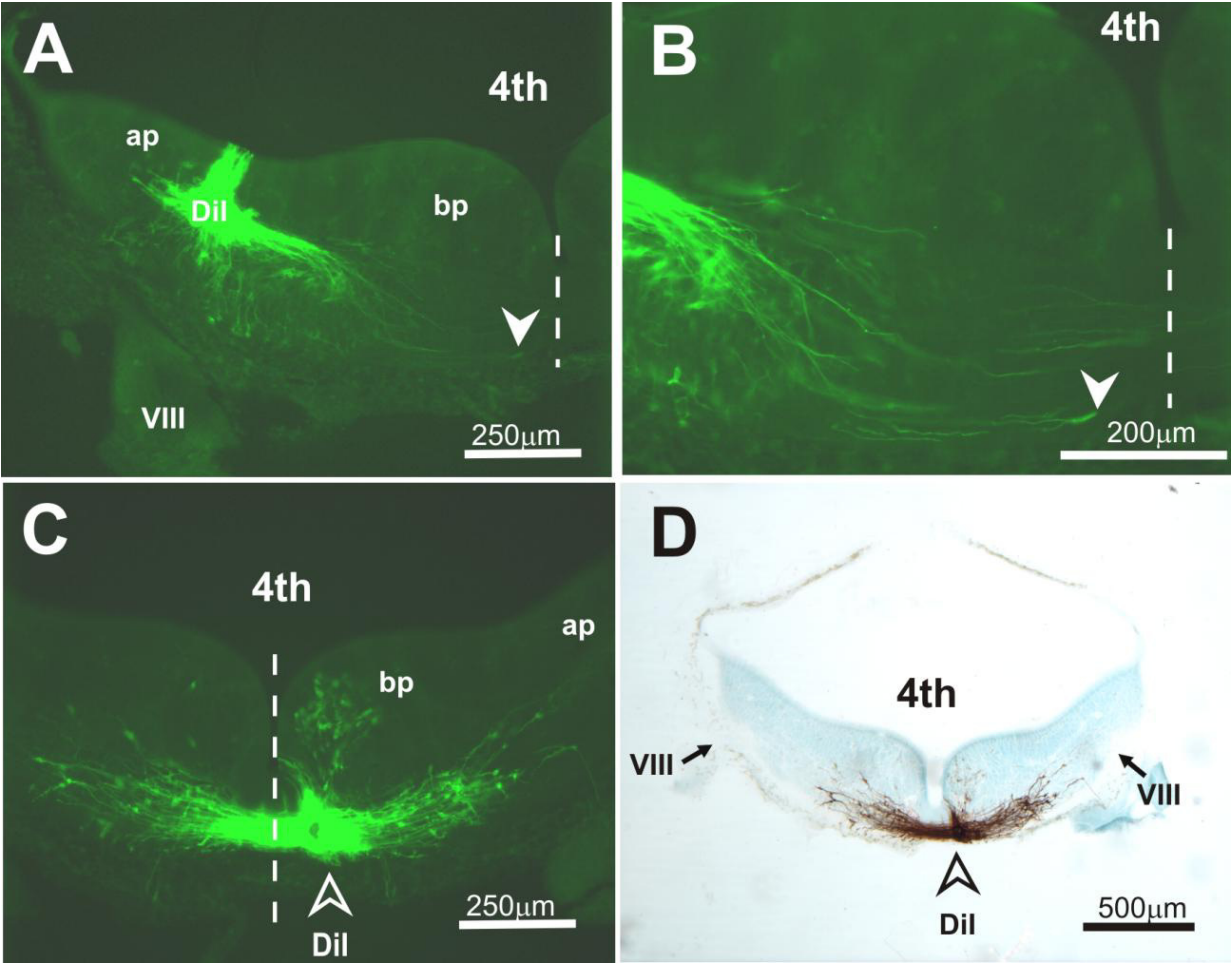
**Preliminary experiments to track the course of labeled fibers following DiI injection into the indicated sites in the hindbrain (E13.5)**. **(A,B) **Transverse section of the hindbrain at the level of the VIIIth cranial nerve root (VIII) showing DiI applied at a site approximately 150 μm into the alar plate and labeled projections coursing in a ventromedial direction towards the midline. Images were as viewed under epifluorescence microscopy with use of an FITC filter. Panel (B) is a magnified view of (A), showing growth cone-like terminals (solid arrowhead) of projection fibers approaching the midline. Dashed line denotes the midline. (C,D) Transverse section as in (A) but showing DiI applied into the floor plate (open arrowhead) for retrograde labeling of neurons. Images were as viewed under epifluorescence microscopy with use of an FITC filter (C), or after photoconversion of DiI labeling into a stable diaminobenzidine reaction product (D). The latter was counterstained with cresyl violet. Most cells that were so labeled are identifiable within the prospective area of the reticular formation; few, if any, labeled cells are observable in the area of the VN. Abbreviations: 4th, fourth ventricle; ap, alar plate; bp, basal plate; cp, cerebellar primordium; V, Vth cranial nerve root; VIII, VIIIth cranial nerve root; r2 to r7, rhombomeres 2 to 7.

Preliminary experiments that targeted DiI to the VN of E13.5 to 15.5 embryos provided confidence as to the location and depth of dye application into the floor of the fourth ventricle (Figures 4 and 5A). DiI-labeled fibers took a ventromedial course towards the floor plate as the growing end approached but had not reached the midline by E13.5 (Figure 5B). This was confirmed by attempts at retrograde labeling of VN neurons by DiI application at the floor plate - few, if any, VN neurons were so labeled in the E13.5 embryo (Figure 5C,D). By application of NeuroVue Maroon to the VIIIth cranial nerve root in combination with DiI to the VN, we attempted to track NeuroVue Maroon-labeled afferents that terminate on DiI-labeled VN neuronal cell bodies or dendrites. Flat-mount of the hindbrain indicated NeuroVue Maroon-labeled afferents reaching DiI-labeled projections (Figure 1E,F). Whether the afferents terminate on dendritic projections of VN neurons or on long dendrites that extend into the VN complex from the reticular formation cannot be deciphered at E13.5. Although this attempt to characterize the VN neurons was inconclusive, targeting DiI to the VN indicated the possibility of mapping out the developing course of commissural projections from the VN.

**Figure 6:**
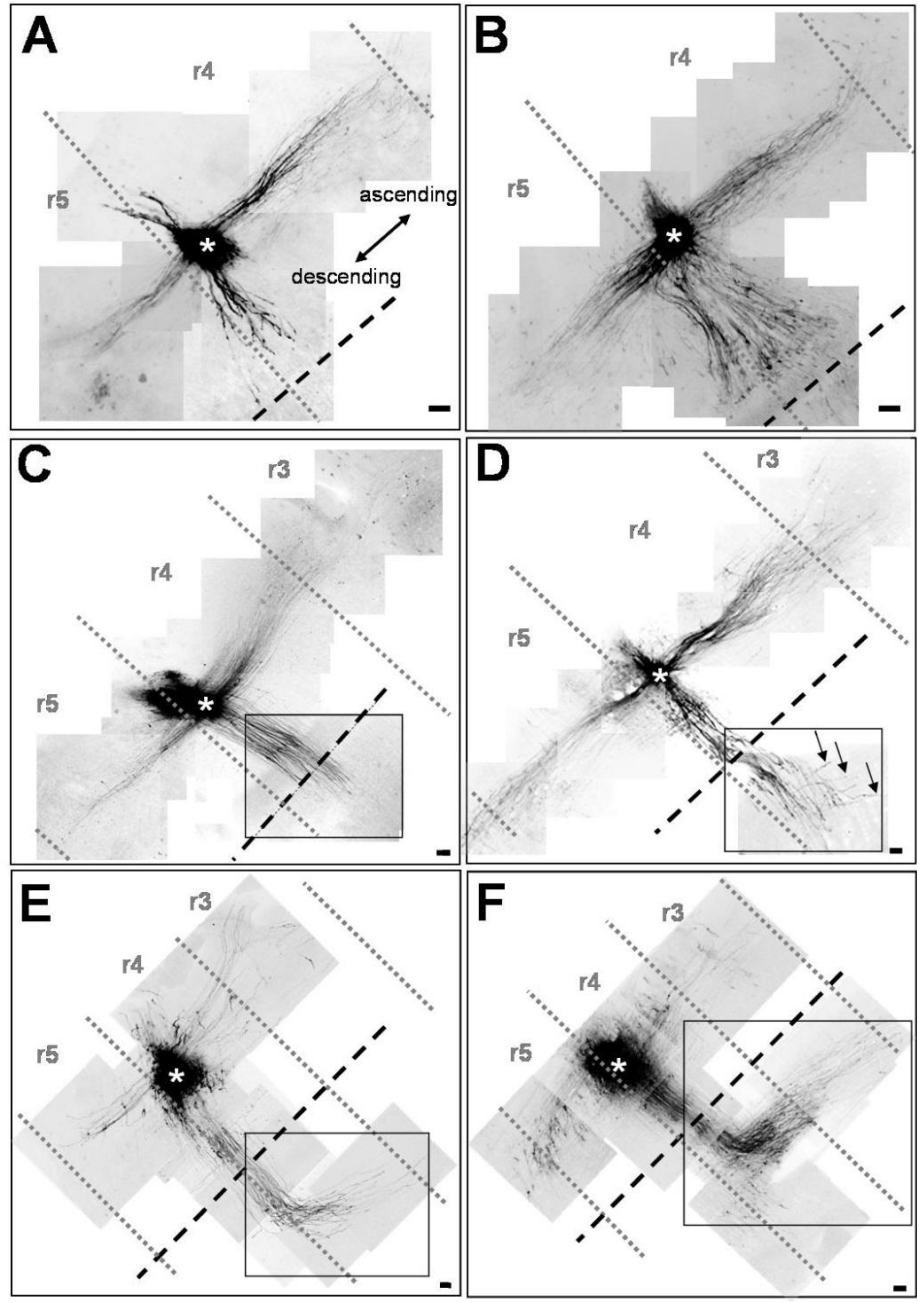
**Gray-scale montages representing DiI-tracked projections from the VN neurons**. **(A,B) **E12.5_(+1 DIV)_; **(C,D) **E13.5_(+1 DIV)_; **(E,F) **E14.5_(+1 DIV). _after the injection of PBS (control) (A,C,F) or Ch^ABC ^(test) (B,D,F). (A) At E12.5_(+1 DIV) _in PBS, projections assumed fascicles as they advanced toward the midline. (B) At E12.5_(+1 DIV) _in Ch^ABC^, many projections were unfasciculated as they approached normal to the midline and a subpopulation crossed the midline. (C) At E13.5_(+1 DIV) _in PBS, projections were fasciculated as they approached and crossed the midline. (D) At E13.5_(+1 DIV) _in Ch^ABC^, pioneer projections advanced normal to the midline as fascicles and beyond towards the contralateral VN. Later projections traversed the enzyme-treated matrix as unfasciculated fibers and deviated from the course of the pioneers (arrows). (E) At E14.5_(+1 DIV) _in PBS, projections advanced as fascicles to the contralateral VN and then turned in the anterior direction. (F) At E14.5_(+1 DIV) _in Ch^ABC^, commissural projections were partly fasciculated and partly not but mostly unfasciculated as they made the anterior turn. Zoomed-in views of boxed areas are shown in Figure 7. Asterisks indicate the site of DiI injection, dotted lines the rhombomere boundaries, and dashed lines the midline. Abbreviations: r, rhombomere;. Scale bar: 100 μm.

**Figure 7:**
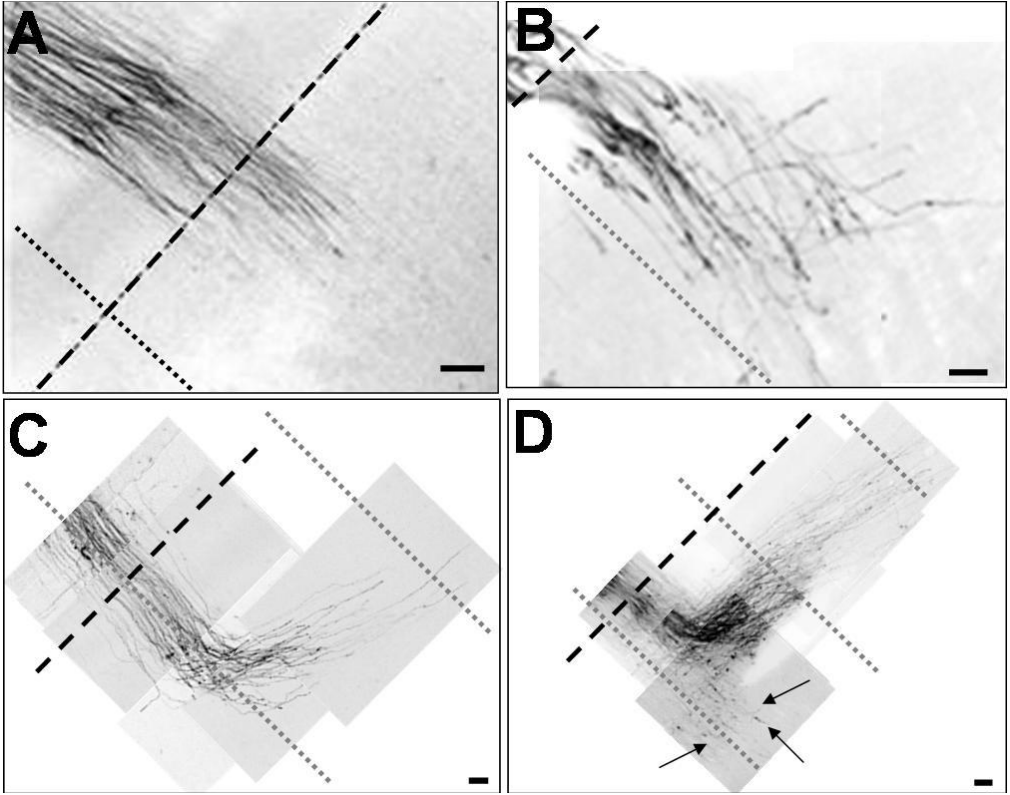
**Zoomed-in views of boxed areas in Figure 6 showing fibers at midline crossing and at the contralateral site**. **(A,B) **E13.5_(+1 DIV)_; **(C,D) **E14.5_(+1 DIV)_; (A,C) control treatment with PBS; (B,D) test treatment with Ch^ABC^. At E13.5 in PBS, fibers advanced as fascicles normal to the the midline. At E13.5_(+1 DIV) _in Ch^ABC^, a subpopulation of unfasciculated fibers projected normal to the midline and crossed to the contralateral side but others deviated from the normal trajectories. At E14.5_(+1 DIV) _in Ch^ABC^, most fibers made the anterior turn but some unfasciculated fibers (arrows) failed to make the turn.. The dashed line indicates the midline, and dotted lines the rhombomerre boundaries. Scale bar: 100 μm.

**Figure 8:**
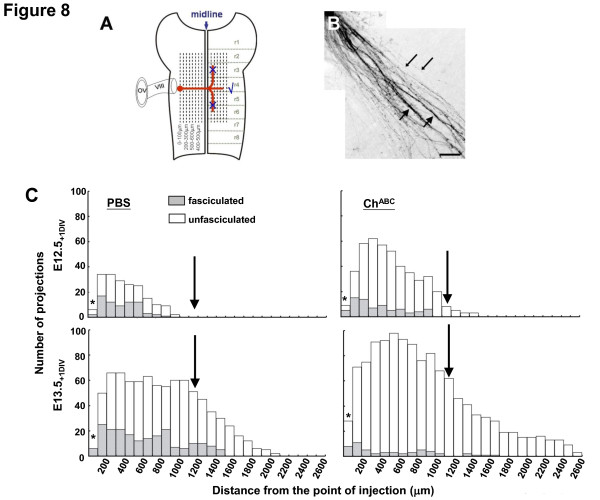
**Quantification of DiI-tracked commissural projections of the VN**. **(A) **Schematic diagram showing overlay of longituinal gridlines against DiI-tracked fiber projections (red) of the hindbrain flat-mounts. The injection site was set as '0' and projections to the right (contralateral) were counted. **(B) **Image of fasciculated fibers (thick arrows) mixed with unfasciculated fibers (thin arrows). **(C) **Histograms of fasciculated and unfasciculated projections against the distance from the DiI injection site in hindbrains treated with PBS (left panels) and with Ch^ABC ^(right panels). Data are representative of five individual sets of experiments at E12.5_(+1 DIV) _and E13.5_(+1 DIV)_. The downward pointing arrow indicates the position of the midline. Asterisks denote regions within 100 μm of the DI injection site where the intensity of dye precluded accuracy of fibre counts.

The changes in commissural projections from the VN as a result of treatment with Ch^ABC ^were then studied with DiI-tracking in respect of projection distance, number of DiI-tracked fibers, fasciculation of DiI-tracked fibers and projection direction. Among E12.5_(+1 DIV) _embryos that had undergone control treatment with heat-inactivated Ch^ABC ^or PBS, vestibular commissures appeared fasciculated and projected in a direction almost normal to the midline (Figure 6A). This was not different from projections found in E13.5 embryos that developed *in utero*. Given the high cost of commercial Ch^ABC^, treatment of stage-matched embryos with PBS was considered an adequate control to test treatment with Ch^ABC^. Results presented are representative of at least five independent experiments in which PBS control and Ch^ABC ^treatments were performed on littermates. Montage images of the VN commissural projections in PBS- versus Ch^ABC^-treated hindbrains are shown in Figure 6 and zoomed-in views of projections that crossed the midline are shown for E13.5_(+1 DIV) _(Figure 7A,B) and E14.5_(+1 DIV) _(Figure 7C,D). Corresponding histograms of the projections, fasciculated and unfasciculated, in the direction of the contralateral VN are shown in Figure 8.

#### Projection direction

Only projections toward the contralateral side were plotted in the histograms. For PBS controls at both E12.5_(+1 DIV) _and E13.5_(+1 DIV)_, the entire population of contralateral fibers projected in a direction normal to the midline. In cases of Ch^ABC^, unfasciculated fibers that deviated from this normal direction were excluded and not represented in the histograms.

Pathway deviation by unfasciculated fibers reflects that Ch^ABC ^acted to expose alternative cues that detract the late projections from the pioneer path. Matrix CS therefore restricts late projections to the path charted out by pioneer projections.

#### Number of DiI-tracked fibers

At E12.5_(+1 DIV) _in PBS, 74% (25/34) of initial projections toward the contralateral side extended as far as 600 μm. Only 10 or fewer extended beyond 600 μm and numbers declined at a rate of 3 fibers per 100 μm. At E12.5_(+1 DIV) _in Ch^ABC^, projections that extended as far as 200 μm were increased by 2 to 3 times compared to the PBS control, totaling 60 fibers and declining at a rate of 2 fibers per 100 μm to reach 30 fibers by 600 to 1,000 μm Some 20 fibers crossed the midline and < 10 projected for 350 μm beyond the midline; the rate of decline across the floor plate (1,000 to 1,300 μm) was 5 fibers per 100 μm.

At E13.5_(+1 DIV) _in PBS, the initial projections (around 60) persisted up to 1,100 μm (approximately the midline). Thereafter, numbers declined at a rate of 5 fibers per 100 μm up to 1,400 μm and then at lower rates until they were at a barely detectable level at 2,100 μm. In Ch^ABC^, from about 70 fibers in the initial 200 to 300 μm, new levels reaching about 90 were maintained up to 700 μm. Numbers dropped at a rate of 7 fibers per 100 μm across the midline to 1,400 μm. Then gradually at a rate of 3 fibers per 100 μm, numbers reached 15 fibers and remained so within 1,900 to 2,100 μm before further declining to the basal level at 2,600 nm. At both stages, the increased numbers on the ipsilateral side reinforce that depletion of matrix CS by Ch^ABC ^activity removed limitations to fiber space such that late projections remain unfasciculated as they traverse the hindbrain matrix toward the contralateral side.

#### Fasciculation of DiI-tracked fibers

At E12.5_(+1 DIV) _in PBS, 30 to 50% of projections within the initial 500 μm were fasciculated and by 700 to 900 μm, fibers were few and mainly unfasciculated. In Ch^ABC^, a declining proportion (42 to 25% of fibers) within the initial 200 μm was fasciculated and an increasing proportion was unfasciculated. From 300 to 700 μm, the proportion of fasciculated fibers remained low at 10 to 11%. Fibers crossing the midline and projecting in the contralateral side were unfasciculated.

At E13.5_(+1 DIV) _in PBS, 30 to 40% of projections remained fasciculated up to 800 μm. Across the midline till 1,400 μm, fasciculated fibers were maintained at 20% of projections but total fiber number declined. By 1,500 μm, fibers were unfasciculated. Hardly any were detectable by 2,100 μm. In Ch^ABC^, > 95% of fibers on the ipsilateral side (within 900 μm) were unfacsiculated. Across the midline till 2,600 μm, fasciculated fibers were minimal, if any. Digestion of matrix CS by Ch^ABC ^therefore removes barriers that otherwise limit follower projections to fasciculate and to conform with the path of pioneer projections.

#### Projection distance

At E12.5_(+1 DIV) _in PBS, the furthest projections were short of the midline by 150 μm. In Ch^ABC^, the furthest projections were 350 μm beyond the midline. At E13.5_(+1 DIV) _in PBS, projections reached as far as 2,100 μm into the contralateral side. In Ch^ABC^, projections reached further into the contralateral side, reaching 2,600 μm. Digestion of matrix CS by Ch^ABC ^therefore removes spatial limitations and promotes growth and advancement of pioneers to regions even beyond the contralateral side.

At E14.5_(+1 DIV)_, in both PBS controls and Ch^ABC^-treated embryos, the distance between the DiI injection site and the midline was apparently shorter than those in E12.5_(+1 DIV) _and E13.5_(+1 DIV) _embryos (Figure 6). This was related to a decrease in the area of the ventricular surface of the neural tube with development and an increase in the thickness of the neural tube due possibly to proliferation of neuroepithelial cells and matrix deposition by neuroblasts. Proper flat-mounts of embryonic hindbrains could hardly be prepared. As a result, fiber projections were at angles inclined from the horizontal but viewed as if flat on the page.

## Discussion

In the present study, our results show that CS moieties in the rat hindbrain matrix (E12.5_(+1 DIV) _and E13.5_(+1 DIV) _) are important for controlling commissural projection of VN neurons at a time when few, if any, fiber tracts have charted out their course in the early hindbrain.

During the development of the hindbrain, segmentation of the neuroepithelium into rhombomeres is important in laying out the framework for correct neuronal patterning [2]. Projection of VN commissures during this period when few fiber tracts exist and the coincidental abundance of CSPGs in the hindbrain matrix suggest a role for this molecule in regulating the commissural projection. Studies of the embryonic chick brain showed CSPG accumulation in the extracellular matrix of rhombomere boundaries [17,18]. The CS moiety has been considered important in restricting cell clusters and preventing them from intermingling with clusters of different cell lineages and thus in maintaining identities of cell clusters in the hindbrain. With the use of immunostaining for the CS-56 epitope and the 2B6 stub epitope following Ch^ABC ^treatment of the hindbrain matrix, we showed that the CS moieties are localized in the extracellular matrix of rhombomeric segments as well as the boundaries of rhombomeres (Figure 3A,E). Cells and their projections can traverse this CS-rich region in the rhombomeres. So, does the restrictive role of CS in rhombomeres apply in such circumstances?

The CS moiety has been implicated in the control of neurite growth and extension. Culture of chick retinal ganglion cells on nitrocellulose filters that have been implanted in a lesioned cerebral cortex to capture CSPGs of the glial scar showed that treatment of the filter with Ch^ABC ^resulted in cultures bearing longer neurites [19]. Treatment of chiasmatic brain slice cultures (E13 to E15, mouse) with Ch^ABC ^resulted in misrouting of retinal ganglion cell projections at the optic chiasma [20]. In the present study, we showed that treatment of hindbrain matrix with Ch^ABC ^in 1-day cultures of rat embryos sped up projection along the commissural route toward the contralateral VN. This effect was evident in both E12.5_(+1 DIV) _and E13.5_(+1 DIV) _embryos - in the former, projections reached further by 800 μm along the commissural route, and in the latter, projections ended up in regions beyond the contralateral VN (Figure 8). Our results suggest that CS moieties prevailing in the early hindbrain matrix play an axon-restrictive role in controlling the speed of projection of the VN commissures such that the terminals can recognize the targets on arrival.

In this work, we found that VN commissural fibers were less fasciculated in hindbrains that had been treated with Ch^ABC ^than those of controls treated with PBS. For assessment of the extent of fasciculation of the VN commissural fibers in 100-μm steps across the hindbrain, we chose fiber diameters of > 5 μm as an arbitrary indication of fasciculation and those ≤5 μm as an indication of non-fasciculation. This cutoff makes it possible for exhaustive assessment of all DiI-tracked fibers at the light microscopic level. Assessment of fibers of ≤5 μm in close-up view revealed that these included three to five single projections that, at times, were separate from each other and, at other times, remained indiscernible. To decipher these would require viewing at the electron microscopic level. This latter approach can only reveal a small portion of the examined area each time and is impractical for assessment of all the labeled VN commissures. Our observation of increase in unfasciculated fibers with Ch^ABC ^treatment of the hindbrain suggests that axon-restrictive CS moieties in the hindbrain matrix induced the late outgrowths or follower projections to join the track of pioneers and thus to fasciculate with each other.

Earlier studies considered CSPG as an entity and did not attempt to distinguish between roles of the CS moiety and those of the core proteins. Thus, such CSPGs as neurocan and phosphacan were found to mediate axonal fasciculation during development of the hippocampal circuitry *in vivo *[21]. In cultures of hippocampal neurons, dorsal root ganglia or habenula nucleus neurons, neurite extension into a CSPG substratum exhibited fasciculation as well [21-23]. These provide evidence for axonal fasciculation as a means to modulate the ability of the growth cone to navigate through restrictive environments or regulate axonal pathfinding during circuit formation.

Our results provide an *in vivo *correlate to the *in vitro *findings of [24] that the CS moieties of neural proteoglycans such as neurocan and phosphacan have roles in the modulation of neuronal adhesion and neurite growth by binding to neural cell adhesion molecules like neuron-glia cell adhesion molecule (Ng-CAM) and neural cell adhesion molecule (N-CAM). Treatment of the hindbrain matrix with Ch^ABC ^when the projecting fibers are in the growing mode not only diminished matrix restriction of cues and guideposts, but can likely interfere with the modulation of molecular interactions involving the CAMs at close axonal contacts. The CAMs on neurons have sites for homophilic binding with cell attached CAMs [25-27], and other sites for binding with neurocan and phosphacan, both the glycosylated form and the Ch^ABC^-treated counterpart [28,29]. Direct binding of neurons with the native or Ch^ABC^-treated neurocan and phosphacan via Ng-CAM and N-CAM was demonstrated [28,29]. We reason that the CS moieties are uniquely poised to fine-tune the balance between adhesion and anti-adhesion for growing axons to navigate along pre-existing axonal trajectories.

The strong CS-56 immunopositivity remaining at the ventricular surface despite the proximity to the infused Ch^ABC ^suggests limited access of the enzyme to CS moieties on the ventricular surface. It is likely that the CSPGs were protected from enzymatic digestion via interaction with other extracellular matrix molecules. In one of the other experiments in our lab, we have shown that the release of semaphorin3A, which binds to CS moieties in the central nervous system [30], could only be achieved when the samples were treated with both Ch^ABC ^and heparitinases, but not with Ch^ABC ^alone (unpublished data). These results support that by interaction with other extracellular matrix molecules, the CS moiety is protected from the action of Ch^ABC ^and presents as residual immunoreactivity to CS-56.

There is a concern that the differences we observed in axonal trajectories with and without Ch^ABC ^treatment is due to increased apoptosis in the embryos, in particular the higher mortality rate among E14.5 embryos in culture (Figure 2). Given that CSPGs bind various chemokines and growth factors (personal communication), Ch^ABC ^cleavage of the CS moiety may result in loss of these factors and therefore increased incidence of cell death. In order to verify that the results we reported are due to Ch^ABC ^cleavage of the CS moiety but not other cellular processes, such as apoptosis, we monitored the development of the embryos by their heartbeat and their growth in size after the *ex vivo *culture. We did not observe any differences in size between the PBS- and Ch^ABC^-treated embryos. Embryos with slow heartbeats or reduced size were discarded at the end of the culture and were not included for DiI tracking of commissural projections from the VN. Moreover, cross-sections of these embryos have been immunostained with NF165 and results showed that the numbers of neurites were similar in both cases (results not shown). Admittedly, these results cannot provide definitive proof that all subpopulations of neurons are equally protected. More experiments have to be performed to reveal the contribution of CSPGs to the survival of subpopulations of neurons.

CS is a well known inhibitory molecule in the nervous system. The high abundance of CSPGs in the glial scar that forms after nervous tissue injury suggests their role in limiting regeneration [31,32]. With the removal of CS by Ch^ABC ^treatment of the glial scar, axonal transgression of the scar was observed [33-39]. This raises the question of how the projections negotiate their way through the CS-rich environment. With rat dorsal root ganglia in culture on aggrecan-laminin substrata, inhibition of neurite extension by the CS moiety of aggrecan was attributed to suppression of integrin signaling and that the inhibition could be reversed by integrin activation [40]. Alternatively, chick dorsal root ganglia culture treated with versican V0/V1 forms revealed concentration-dependent neurite responses that ranged from rapid retraction and deflection to defasciculation and growth arrest [41]. Our work is the first to demonstrate the role of CS moieties in the early hindbrain matrix in controlling the pattern of projection of VN commissures for timely arrival at the contralateral target. Further study on various CS isoforms may give new insights into mechanisms by which the CS moiety can fine-tune adhesion versus anti-adhesion signaling at the growth terminal to allow navigation along axonal trajectories in early hindbrain development.

## Competing interests

The authors declare that they have no competing interests.

## Abbreviations

Ch^ABC^: chondroitinase ABC; CAM: cell adhesion molecule; CS: chondroitin sulfate; CSPG: chondroitin sulfate proteoglycan; Dulbecco's modified Eagle's Medium; E: embryonic day; N-CAM: Neural cell adhesion molecule; Ng-CAM: neuron-glia cell adhesion molecule; PBS: phosphate-buffered saline; VN: vestibular nucleus.

## Authors' contributions

JCFK performed chondroitinase treatment of rat embryos in culture, analyzed mmunohistochemical results and DiI-tracked fiber projections, and drafted the manuscript. YLY and WKL carried out embryo culture studies, including DiI tracking of commissural projections in the embryonic hindbrain. FXZ performed initial fiber-tracking experiments that standardized the DiI application site in the embryonic hindbrain. JWF participated in the conception of the study and interpretation of chondroitinase activity in the hindbrain. YSC participated in planning of the experiments, analysis and interpretation of fiber projections, and the writing of the manuscript. DKYS conceived of the study, designed and coordinated the experiments, and wrote the manuscript. All authors read and approved the final manuscript.
